# Lmo0171, a Novel Internalin-Like Protein, Determines Cell Morphology of *Listeria monocytogenes* and Its Ability to Invade Human Cell Lines

**DOI:** 10.1007/s00284-014-0715-4

**Published:** 2014-10-17

**Authors:** Radosław Stachowiak, Tomasz Jagielski, Katarzyna Roeske, Olga Osińska, Paweł Gunerka, Jarosław Wiśniewski, Jacek Bielecki

**Affiliations:** Department of Applied Microbiology, Faculty of Biology, University of Warsaw, Warsaw, Poland

## Abstract

Internalins comprise a class of *Listeria monocytogenes* proteins responsible for activation of signalling pathways leading to phagocytic uptake of the bacterium by the host cell. In this paper, a possible role of Lmo0171—a new member of the internalin family was investigated. Disruption of the *lmo0171* gene resulted in important cell morphology alterations along with a decrease in the ability to invade three eukaryotic cell lines, that is Int407, Hep-2 and HeLa and diminished adhesion efficiency to int407, thereby suggesting bifunctionality of the newly characterised Lmo0171 internalin.

## Introduction

*Listeria monocytogenes* is a facultative intracellular pathogen and the causative agent of a food-borne disease listeriosis. The disease primarily affects pregnant women, newborns, older adults, and people with an impaired or weakened immune system. The clinical manifestations of human listeriosis range from influenza-like symptoms and gastroenteritis to septicemia, meningitis, encephalitis, perinatal infections, and abortion [5, 10, 12].

*L. monocytogenes* is not only able to invade macrophages but also, with the aid of several adhesins and invasins, non-phagocytic cells [8]. In such case, adhesion and internalisation are mediated mainly through a group of proteins called internalins (Inl), a common feature of which is the presence of the N-terminal leucine-rich repeat domain (LRR) composed of tandem repetitions of 20-22 amino acids, with a large number of leucine or isoleucine residues. The LRR domain is known to be involved in various processes such as adhesion, signalling or ligand-receptor interactions [3]. Most of the so far identified internalins harbour additional domains, such as the inter-repeat region (IR), essential for the binding and internalisation of *Listeria* into a host cell or the C-terminal LPXTG motif, which anchors the protein to peptidoglycan of the bacterial cell wall. Only four internalins, namely InlC, Lmo2027, Lmo2445, Lmo2470 are secreted to culture supernatant, with all the rest being cell wall-attached proteins [2].

Two internalins, designated InlA and InlB have been shown to play a critical role in *L.* *monocytogenes* intracellular invasion. Those internalins are most extensively studied and characterised. Whereas InlA binds to the host cell receptor glycoprotein E-cadherin and is responsible for the uptake of bacteria into intestinal enterocytes, InlB interacts with the hepatocyte growth factor receptor (Met), the receptor for the complement factor C1q-R (gC1qR), and glycosaminoglycans inducing bacterial invasion of different cell types, including hepatocytes, fibroblasts, and epithelial cells [1, 16]. The function of eight other internalins designated InlC—InlJ as virulence factors was proved in vivo [4, 20, 21].

The aim of this study was to investigate the role of Lmo0171, a new listerial internalin-like protein during the early stages of listerial infection. This was achieved by constructing *L. monocytogenes* strain carrying disruption of the *lmo0171* gene and by assessing the abilities of the mutant strain to adhere and invade human cell lines Int407, Hep-2 and HeLa.

## Materials and Methods

### In Silico Analysis

Nucleotide sequences of the *lmo0171* gene, as well as the amino acid sequence of its protein product were analysed bioinformatically. Homology of the Lmo0171 to other known internalins was analysed by the BLASTP alignment, while conserved domains of the protein were predicted by conserved domain search service (CD Search). Both programmes are available online at http://www.ncbi.nlm.nih.gov.

### Bacterial Strains and Growth Conditions

Strains of *L. monocytogenes* EGD (wild type) [17] and EGD mutant strain designed OG1 (*lmo0171*::pKSV7) were grown in brain heart infusion (BHI) broth at 37 and 42 °C. *Escherichia coli* strain DH5α was grown in lysogeny broth (LB). When necessary, media were supplemented with the appropriate antibiotics at the following concentrations: ampicillin, 100 µg mL^−1^ and chloramphenicol, 10 µg mL^−1^.

### Cloning, Gene Disruption and Generation of *lmo0171* Mutant Strain

**Fig. 1:**
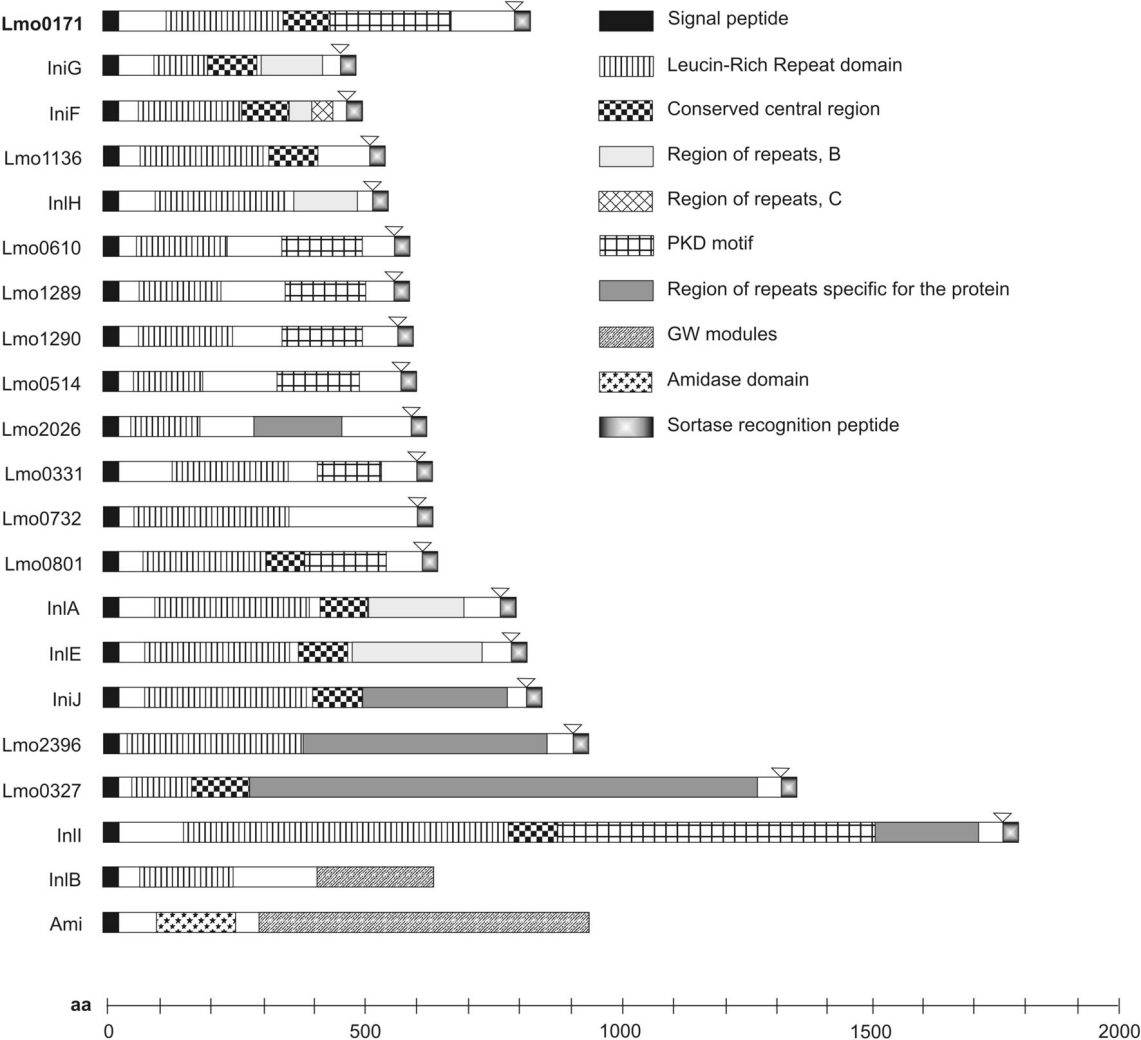
Schematic representation of BLASTP analysis of homology of Lmo0171 and other known or hypothetic internalins. Modular organization of analyzed proteins was represented by different filling patterns

Strain carrying disruption of the *lmo0171* gene was obtained as follows. The 488-bp fragment of the *lmo0171* gene was PCR-amplified with the primers p171L (5′-TCTCTAGAGCATTTAACAGCGT-3′) and p171R (5′-TCAATCGTTAATTTTCGGTAGC-3′). The PCR product was then digested with *Xba*I restriction enzyme and cloned into a *Xba*I site, as a sticky end ligation, of the temperature-sensitive shuttle plasmid pKSV7 [22]. After verifying the construction element by sequencing with primers pksvL (5′-GGGTAACGCCAGGGTTTTCCCAGTC-3′) and pksvR (5′-GCTTCCGGCTCGTATGTTGTGTGGA-3′), the resultant vector pKSV7::*lmo0171* was electroporated into *L.* *monocytogenes* EGD strain, according to a protocol described elsewhere [18]. Transformed cells were plated on BHI with chloramphenicol at 30 °C followed by three passages at 42 °C to ensure integration of the pKSV7 into the *L.* *monocytogenes* chromosome by homologous recombination. Integration of the plasmid was confirmed by PCR reaction with primers pksvL (5′-GGGTAACGCCAGGGTTTTCCCAGTC-3′) and p0171BsL (5′- TGGAAGACATAAACAAAGCT -3′), generating a 1-kb fragment for the OG1 strain and no product for the wild-type strain (Fig. 1a). Those results were supported by the genomic DNA sequencing with the aforesaid pair of primers (i.e. pksvL and p0171BsL). Subsequently, total RNA from both the recombinant OG1 and EGD strains was isolated using Total RNA isolation kit (A&A Biotechnology, Poland) and subjected to RT-PCR using primers pRT171L (5′-CCAGTGAAGAAGCTGCAACT-3′) and pRT171R (5′-TTCGGTAGCCGAGTTAGTTC-3′), amplifying a 720-bp fragment of the *lmo0171* gene. RT-PCR of neighbouring downstream *lmo0174* and upstream *lmo0170* genes was also performed using primer pairs pRT174L (5′-ATCCAGCAGAAATAAAAGAAA-3′) and pRT174R (5′-CTTTCCAATGCCTTGTAC-3′), and pRT170L (5′-CTTGGATAGCCGTAGACGA-3′) and pRT170R (5′-TTCTGCTGGAAGTTCGTCTG-3′), respectively to prove stability of their transcription.

### Phenotypic Analysis, Haemolytic Activity, Stress Response

The growth of the OG1 and EGD *L. monocytogenes* strains was assessed by measuring optical density of the refreshed bacterial cultures and by determining CFU count after plating serial dilutions on LB agar plates. Haemolytic activity of the OG1 strain was assayed essentially as previously described [23]. Briefly, sheep blood was washed three times with PBS, and suspended to a final concentration of 20 % in PBS (pH 7.4). 20 μL of supernatant was added to a final volume of 1 mL 2 % erythrocyte suspension in PBS. The solution was incubated for 30 min at 37 °C, and then centrifuged for 3 min at 150×*g*. The released haemoglobin was measured spectrophotometrically at 410 nm. BHI medium was used as a negative control and an erythrocyte sample lysed with 0.01 % SDS was positive control. Nutritive stress response was examined by cultivating the EGD and OG1 strains at different temperatures (at 4, 16, 30, 37, and 42 °C) and at various pH levels (5.5 and 7.5), followed by the measurement of absorbance at 600 nm and observation of the viability of the bacteria after plating them on LB agar.

### Cell Culture

Three examined cell lines namely Int407 (ECACC 85051004), Hep-2 (ECACC 86030501) and HeLa (ECACC 93021013) were maintained at 37 °C in 5 % CO_2_ atmosphere. Int407 and HeLa cells were cultured in DMEM medium and Hep-2 cells were grown in MEM medium (Gibco, San Diego, United States). Both media were supplemented with 10 % fetal bovine serum (Gibco, San Diego, United States), 100 IU/mL penicillin and 100 μg/mL streptomycin. For all experiments, cells were cultured on glass coverslips placed in Petri dishes containing appropriate tissue culture medium. Prior to adhesion and invasion experiments, cells were washed with fresh culture medium not containing antibiotics to prevent their negative impact on infecting *L.* *monocytogenes* cells.

### Adhesion and Invasion Assays

Both adhesion and invasion assays were carried out with three cell lines listed above. Gentamycin protection assay was performed as described elsewhere [6] to asses invasiveness. Shortly, overnight cultures of *L. monocytogenes* strains were split 1:20 and cultured until reaching OD_600_ of 0.85–0.95 (late logarithmic growth phase). Subsequently, bacterial cells were harvested at 8,000 RPM for 5 min, washed twice in PBS pH 7.4 and resuspended in the same buffer to the appropriate density. Eukaryotic cells were then subjected to infection at MOI of 100:1 bacteria per cell in DMEM medium and bacteria were allowed to adhere for 60 min at 37 °C in 5 % CO_2_ atmosphere. Cell monolayers were washed by pipetting with 3 changes of phosphate-buffered salin (PBS) to remove non-adhered *L.* *monocytogenes*. This point was considered as time 0 for all analyses. The medium was replaced with DMEM containing 100 μg/mL gentamicin and incubated for 60 min to kill extracellular *L. monocytogenes* (time 1). The gentamnycin protection assay was performed as described elsewhere [16]. At appropriate time points, the above infection sequence was interrupted in order to assay for bacterial adhesion or invasion as described below. In cell adhesion assay, at time 0, monolayers infected with FITC labelled bacteria were either fixed and analyzed by fluorescence microscopy or dispersed and plated for determination of colony forming units (CFUs). Adhesion by microscopic analysis was determined from at least three randomly picked fields (field size of 760.9 µm per 570.7 µm) from each of at least three infected monolayers per strain tested. For adhesion by CFU counts, the entire monolayer covering each coverslip was dispersed by incubation in 400 μL of PBS with 0,1 % Triton X-100. Appropriate dilutions of the suspension were plated onto BHI-agar. To determine invasiveness, at 1 h post-infection (time 1), eukaryotic cells were disrupted by Triton X-100 as described above, diluted in PBS pH 7.4 and plated onto BHI-agar plates to determine number of intracellular bacteria. Differences in the measured variables between the experimental and control groups were assessed with the Student’s *t* test. Statistical significance was set at *P* < 0.05.

### Visualisation and Observation

For bright-field microscopy, coverslips were stained with Diff-Quik (American Scientific Products) and mounted with Permount mounting medium (Fisher Scientific). Microscopic observation and microphotographs were done in an Olympus IX70 microscope and the subsequent analysis was performed with ImageJ software. Cells size and number were determined by examining microscopic images with DP-Soft analySIS^®^ software.

## Results

### Description and Mutagenesis of the *lmo0171* Gene

*In silico* analysis revealed that the 2,499-bp *lmo0171* gene (GeneBank accession number: CAC98386.1) encodes a protein, which does not have an equivalent in *Listeria innocua*. As previously described, the *lmo0171* gene expression is PrfA-independent [15]. The product of the *lmo0171* gene, a 832-amino-acid protein (GeneBank accession number: NP_463704.1), shows a significant homology between various strains of *L.* *monocytogenes.* The BLASTP analysis of its homology to other known internalins revealed that Lmo0171 has similar domain construction (Fig. 1). Within the Lmo0171 protein, the following conserved domains were found: the LPXTG-motif cell wall anchor domain, the N-terminal LLR domain consisting of 13 repeats, and the C-terminal LRR domain. Moreover, the presence of three PKD domains, as well as single MucBP and BIG-3 domains were demonstrated.

**Fig. 2:**
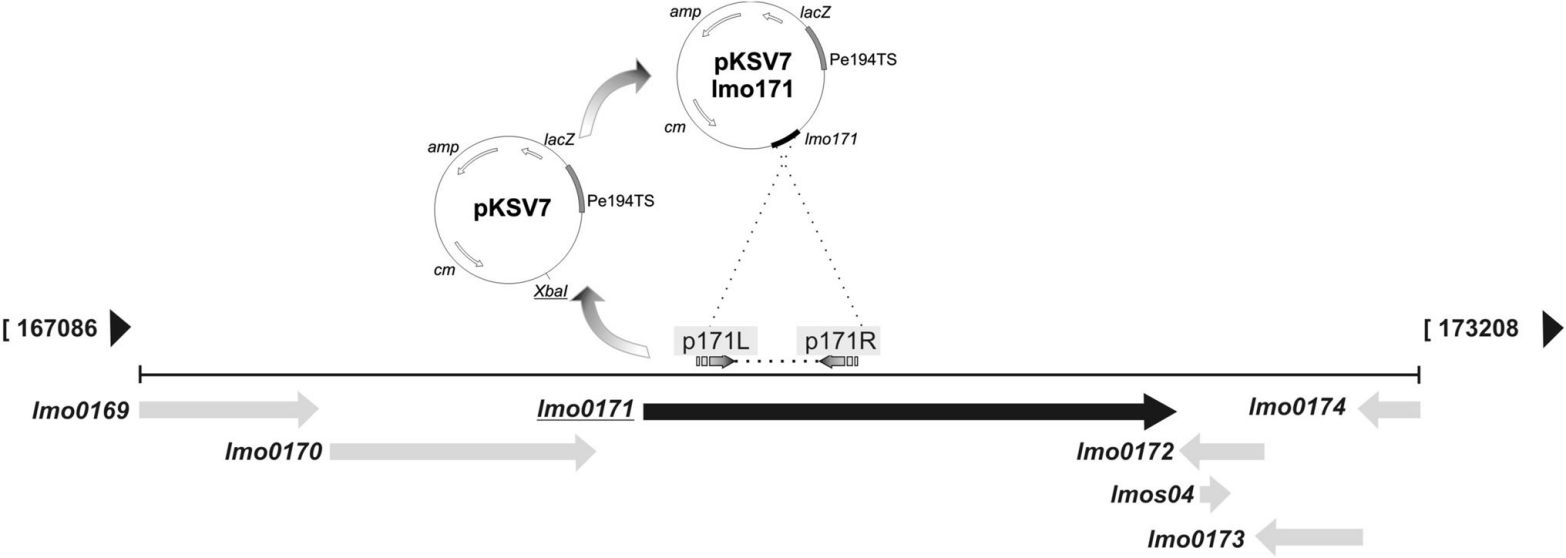
Genomic localization of *lmo0171* gene and a model of used mutagenization. The *lmo0171* fragment was amplified with primers p171L and p171R and cloned into *Xba* I site of pKSV7 plasmid. The resultant vector was electroporated to the competent *L. monocytogenes* cells and chloramphenicol-resistant transformants were selected. Several restriction enzyme cleavage sites and the locations of *lmo0171* and other essential sequences are indicated (*amp*—ampicillin resistance gene, *cm*—chloramphenicol resistance gene, *lacZ*—beta-galactozidase gene, pE194TS—thermosensitive replication origin)

**Fig. 3:**
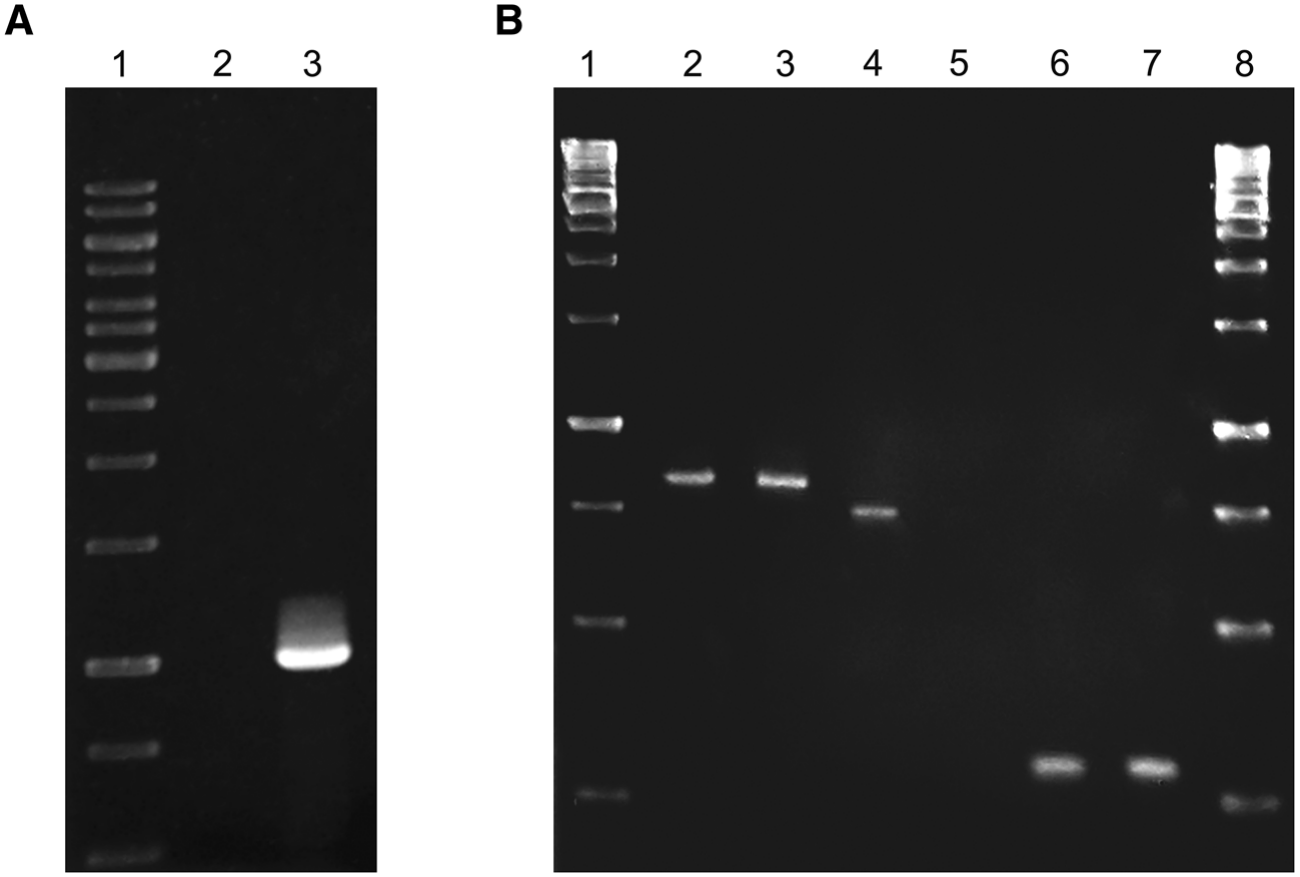
**a** Results of PCR amplification of the lmo0171 gene using chromosomal DNA of *L. monocytogenes* EGD (2) and OG1 (2) strain (3); GeneRuler 1 kb DNA Ladder (1, 8). **b** Results of the RT-PCR for the *lmo0170* (2 = 3), *lmo0171* (4-5), *and lmo0174* (6-7) genes, using total RNA of *L. monocytogenes* EGD (2, 4, 6) and OG1 (3, 5, 7) stran; GeneRuler 1 kb DNA Ladder (1, 8)

To clarify the role of the Lmo0171 protein, *L. monocytogenes* strain OG1 carrying an insertional deletion of the *lmo0171* gene was obtained. Figure 2 shows the scheme of *lmo0171* genomic localization and used mutagenesis strategy. Upon PCR with primers specific to *lmo0171* and pKSV7, a 1-kb fragment was demonstrated for the OG1 strain but not for the wild-type strain (Fig. 3a). As shown in Fig. 3b, transcription of the *lmo0171* gene in the OG1 strain was disrupted by insertion. Analysis of the *lmo0171* gene context reveals two genes: the *lmo0170* and *lmo0174* and two pseudogenes: the *lmo0172* and *lmo0173*. The *lmo0170* and *lmo0174* gene transcripts (775-bp and 274-bp, respectively) were present in both strains, indicating that there was no polar effect due to the *lmo0171* gene insertion in the mutant. In order to assess the stability of the construct, three passages were carried out.

### Morphological Characterisation of the *lmo0171* Mutant

No differences in the colony morphology, growth rate or haemolytic activity between the *L. monocytogenes* OG1 and EGD strains were observed. Likewise, no significant differences were noted for the two strains in their growth rates at different temperatures and pH levels.

**Fig. 4:**
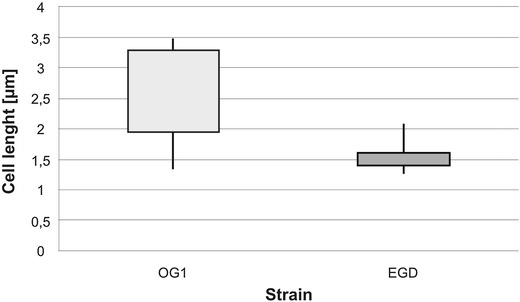
Differences in size of OG1 mutant strain and EGD wild-type strain cells. The bacterial cells were analyzed in an Olympus IX70 microscope and the subsequent analysis was performed with ImageJ software (analySIS^®^, DP-Soft, United States)

**Fig. 5:**
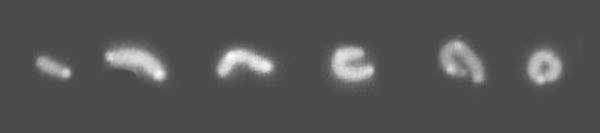
Various morphological forms observed at microphotographs FITC-labelled cells of *L. monocytogenes* OG1 strain in logarithmic growth phase. The cells were observed as typical rod-shaped, but also as bent or curved rods (V-, U-shaped), comma-shaped, and almost circular

The only apparent difference between the wild type and mutant strain concerned the shape of bacterial cells. The cells of the OG1 strain were much longer (1.94 ± 0.61 μm to 3.28 ± 0.20 μm) than those of the EGD strain (1.40 ± 0.14 to 1.60 ± 0.47 μm) (Fig. 4). Furthermore, the OG1 population was morphologically heterogeneous. The OG1 strain cells assumed a variety of appearances, ranging from rods with bent V- or U-shaped ends to almost circular forms, while the wild-type EGD strain grew as typical rod-shape cells. As shown in Fig. 5, at least part of the OG1 strain cells displayed a coma, helical and single turn spiral shape that is other than a typical rod shape. A small part (ca. 2–3 %) of the *lmo0171* mutant strain cells was twisted into spirals.

### The Role of Lmo0171 Internalin in Virulence

**Fig. 6:**
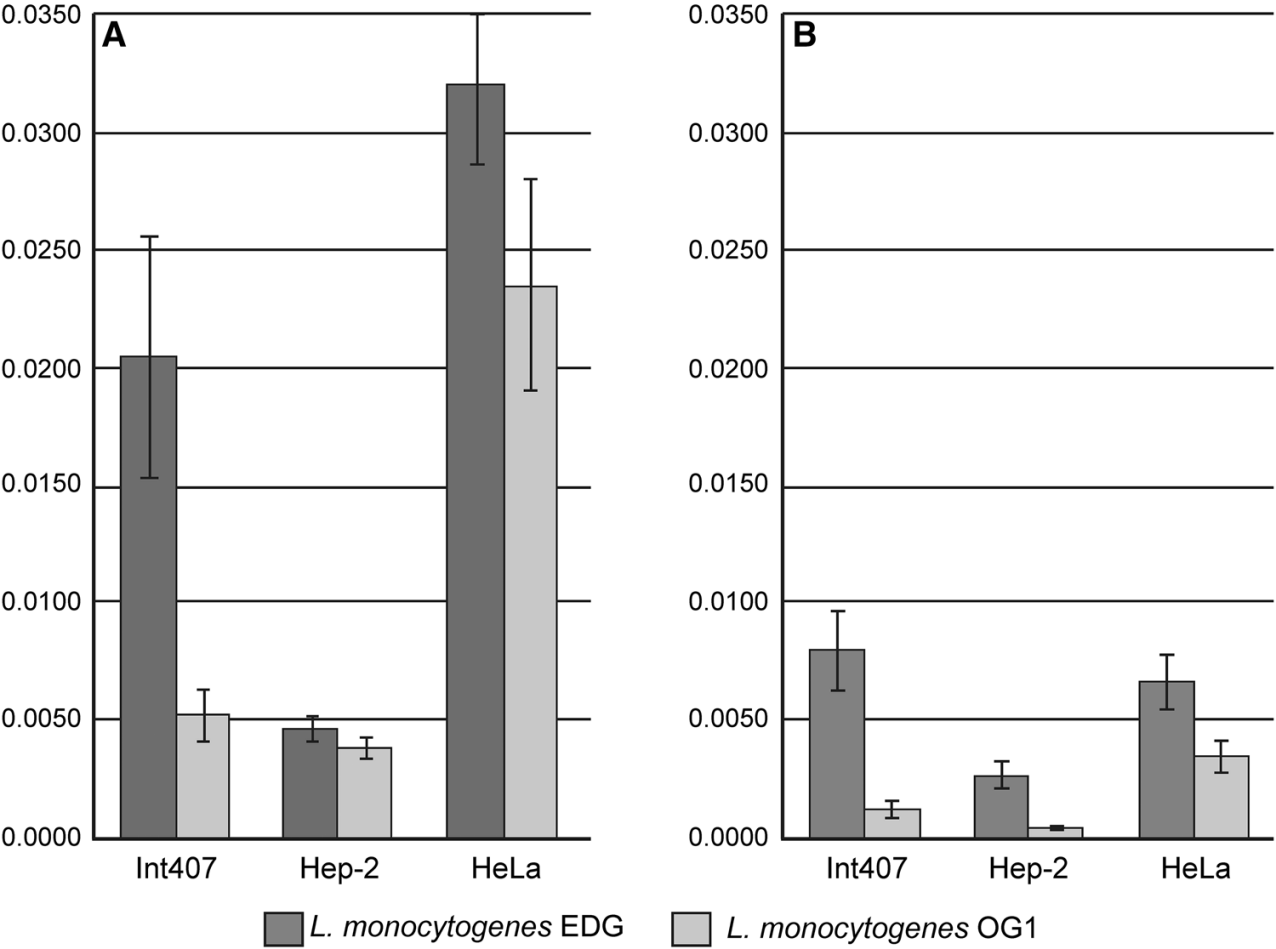
Percent of adhesion (**a**) and entry (**b**) of *L.* *monocytogenes* EGD i OG1 strains into various cell lines. This graph summarises three independent experiments

The ability of the two *L. monocytogenes* strains tested to adhere to the eukaryotic cells was shown to be dependent on the expression of the Lmo0171 on bacterial surface. The adherence of the OG1 strains, defined as the percentage of adhered bacilli on the Int407 epithelial cells, was calculated at 29.8 % ±4.21 of the value of the wild-type strain. Interestingly, both mutant and wild-type strains showed similar rates of adhesion to the cells of other two types (Hep-2 and HeLa) (Fig. 6a).

As for the capacity of invasion, that of the mutant strain was decreased, as compared with the wild-type strain. The decrease of the invasion level was revealed for all the examined cell lines (Fig. 6b). The highest, over 7-fold, drop-off was observed for the Int407 cell line, while the invasion to the Hep-2 and HeLa cells was reduced by 5- and 2-fold, respectively.

## Discussion

In this study, the possible role of the Lmo0171 in the *L.* *monocytogenes* invasion into human cells was investigated. *In silico* analysis revealed that the internalin-like protein Lmo0171 has a domain structure characteristic of other internalins described. Lmo0171 is a putative surface protein bound covalently to cell wall by the sortase SrtA, which recognises the LPXTG motif of the internalin. The N-terminal region of the peptide contains the LRR domain comprising 13 repeats. Similarly to the structure of other internalins (InlA, InlB, InlG, InlH, InlE, and InlF), the LRR domain of Lmo0171 is flanked at its C–terminus by a conserved LRR-adjacent domain. Three PKD repeat domains, MucBP domain and BIG-3 domain were also identified. However, the roles of these domains are unknown. The PKD domain of Lmo0171 was just the same as that of IniI internalin. Whereas domains MucBP and BIG-3 related to Lmo2026 internalin, which is involved in listerial cerebral infection [2]. Analysis of the molecular organization of the Lmo0171 helped to predict participation of this protein in the pathogenesis process.

The presence of a PCR product in the RT-PCR assay, with a wild-type EGD strain chromosome as a template, confirmed that the *lmo0171* gene coding for the novel internalin is not a pseudogene. In the mutant OG1 strain, the RT-PCR product was not observed, indicating that transcription of the *lmo0171* gene was blocked due to the insertional mutagenesis performed with the pKSV7 plasmid. Similar expression level of the *lmo0170* and *lmo0174* genes in both strains, as assessed by RT-PCR, proved that the insertion did not interfere with the neighbouring genes expression.

A decreased level of the OG1 mutant strain invasiveness to all the cell lines used in the study supported the results of the *in* *silico* analysis and confirmed the role of Lmo0171 in bacterial entry to the host cells. A similar phenomenon was previously described for the already known invasion mutants, such as *L. monocytogenes* EGD strains lacking main internalin genes (Δ*inlA*, Δ*inlB*) [13]. The diminished ability of the OG1 strain to enter three different cell lines (Int407, Hep–2 and HeLa) suggests that Lmo0171 is a non-tissue-specific invasin. This is, however, in contrast to past research on internalins, indicating for example specificity of InlA toward the Caco-2 cell line [9]. On the other hand, a lowered adhesion efficiency was observed for the OG1 strain, only when Int407 cells were used. This may speak in favour of different levels of cooperation between Lmo0171 and receptors on the surface of different cell types. It is likely that constant level of adhesion to Hep-2 and HeLa cells is a consequence of the activity of main internalins as was previously observed for internalins InlC-E [4].

Lmo0171 depletion in the OG1 strain resulted in notable changes in bacterial cell morphology. This indicates that the protein may be a part of the cell shape regulatory system which is involved in stabilising the cell wall of most bacteria. The major component of the bacterial cell wall is peptidoglycan, which is responsible for shape determination and osmotic stability. Peptidoglycan polymerization is performed by multienzymatic complexes that include mainly enzymes of two classes—glycosyltransferases, D,D-transpeptidases and hydrolases. To inspect whether these enzymes were affected in the OG1 strain, its cells were treated with penicillin, which inhibits the formation of peptidoglycan cross-links by binding to the enzymes controlling this process. However, the effect of antibiotic treatment on the cell morphology was far different from the morphological changes in penicillin untreated cells (data not shown), indicating that changes in the OG1 strain cell shape are not the result of improper peptidoglycan crosslinking.

It is plausible that Lmo0171 interacts with other proteins within the multipeptide complexes involved in the cell-shape regulation or that it stabilises the cell wall structure. The mechanisms behind the maintenance of bacterial cell-shape remain ill-defined. A few studies suggested that the peptidoglycan-synthesising enzymes might be attached to internal filamentous scaffolds of FtsZ, MreB or Mbl proteins, whose geometric distribution determines the cylindrical form of bacteria. These proteins are thought to be required not only for cell shape determination, but also chromosome segregation or polar protein distribution among many species [11, 14]. It is likely that MreB and Mb1 direct the localization of penicillin-binding proteins (PBPs) and peptidoglycan precursor synthetic enzymes to specific sites of cell wall synthesis.

FtsZ and PBP5 work together to generate the normally uniform, unbranched shape of *E. coli*, while PBPs 4, 6, and 7 play auxiliary roles [24]. Mutants lacking PBPs often adopt abnormal morphology. Morphological aberrations observed in the *lmo0171* mutant may suggest that, similarly to PBPs, Lmo0171 participates in regulation of cell morphology by inducing microtubular cytoskeleton rearrangements.

Another explanation of how Lmo0171 may influence the cell shape may be colocalization of the novel internalin with complex of enzymes involved in peptidoglycan polymerization. Filamental structures serve as scaffolding for the proteins that regulate cell wall synthesis. For instance, in *Bacillus subtilis*, in addition to FtsZ, at least another five proteins, that is FtsA, DivIB, FtsL, DivIC, and PBP2B have been identified as part of the division machinery. The cell division starts from the septal ring formation by FtsZ, with subsequent recruitment of DivIB, DivIC and FtsL. PBP2B responsible for late stages of peptidoglycan synthesis assembles as the last one, depending on the topology of other proteins [7]. In this scenario, the lack of Lmo0171 could provoke localization of reconstruction machinery only on the one side of the cell, thus leading to asymmetry in cell wall growth and curvature towards the shorter cell wall.

In conclusion, two findings of the study are of particular importance. First, the invasion of *L.* *monocytogenes* into the host cells depends, at least to some extent, on the presence of Lmo0171. Second, there is a relationship between expression of the Lmo0171 and bacterial cell shape. The results of this study suggest that Lmo0171 is a bifunctional protein acting both on the cell surface, through mediating adhesion and internalisation, and in the cell cytosol, through mediating cytoskeleton rearrangements. It is not the only listerial protein to display such a bifuncionality. It was demonstrated for the first time for p60 protein (also known as Iap and CwhA) which displays characteristics, as both names appropriately suggest, of Invasion associated protein (Iap) and Cell wall hydrolase (CwhA) [19]. To clarify the exact role of the Lmo0171 protein in the *L. monocytogenes* life cycle, further studies are needed.

## Abbreviations

InlA: Internalin A
InlB: Internalin B
LRR: Leucine-rich repeat

## References

[1] Bierne H, Cossart P (2002). InlB, a surface protein of *Listeria monocytogenes* that behaves as an invasin and a growth factor. J Cell Sci.

[2] Bierne H, Cossart P (2007). *Listeria monocytogenes* surface proteins: from genome predictions to function. Microbiol Mol Biol Rev.

[3] Bierne H, Sabet C, Personnic N, Cossart P (2007). Internalins: a complex family of leucine-rich repeat-containing proteins in *Listeria monocytogenes*. Microbes Infect.

[4] Dramsi S, Dehoux P, Lebrun M, Goossens PL, Cossart P (1997). Identification of four new members of the internalin multigene family of *Listeria monocytogenes* EGD. Infect Immun.

[5] Drevets DA, Leenen PJ, Greenfield RA (2004). Invasion of the central nervous system by intracellular bacteria. Clin Microbiol Rev.

[6] Elsinghorst EA (1994). Measurement of invasion by gentamicin resistance. Methods Enzymol.

[7] Feucht A, Lucet I, Yudkin MD, Errington J (2001). Cytological and biochemical characterization of the FtsA cell division protein of *Bacillus subtilis*. Mol Microbiol.

[8] Finlay BB, Cossart P (1997). Exploitation of mammalian host cell functions by bacterial pathogens. Science.

[9] Gaillard JL, Berche P, Frehel C, Gouin E, Cossart P (1991). Entry of *L. monocytogenes* into cells is mediated by internalin, a repeat protein reminiscent of surface antigens from gram-positive cocci. Cell.

[10] Hof H (2003). History and epidemiology of listeriosis. FEMS Immunol Med Microbiol.

[11] Kroos L, Maddock JR (2003). Prokaryotic development: emerging insights. J Bacteriol.

[12] Lecuit M (2007). Human listeriosis and animal models. Microbes Infect.

[13] Lingnau A, Domann E, Hudel M, Bock M, Nichterlein T, Wehland J, Chakraborty T (1995). Expression of the *Listeria monocytogenes* EGD inlA and inlB genes, whose products mediate bacterial entry into tissue culture cell lines, by PrfA-dependent and -independent mechanisms. Infect Immun.

[14] Margolin W (2000). Themes and variations in prokaryotic cell division. FEMS Microbiol Rev.

[15] McGann P, Ivanek R, Wiedmann M, Boor KJ (2007). Temperature-dependent expression of *Listeria monocytogenes* internalin and internalin-like genes suggests functional diversity of these proteins among the listeriae. Appl Environ Microbiol.

[16] Mengaud J, Ohayon H, Gounon P, Mege RM, Cossart P (1996). E-cadherin is the receptor for internalin, a surface protein required for entry of *L. monocytogenes* into epithelial cells. Cell.

[17] Murray EGD, Webb RA, Swann MBR (1926). A disease of rabbits characterised by a large mononuclear leucocytosis, caused by a hitherto undescribed bacillus *Bacterium monocytogenes* (n. sp.). J Pathol Bacteriol.

[18] Park SF, Stewart GS (1990). High-efficiency transformation of *Listeria monocytogenes* by electroporation of penicillin-treated cells. Gene.

[19] Pilgrim S, Kolb-Maurer A, Gentschev I, Goebel W, Kuhn M (2003). Deletion of the gene encoding p60 in *Listeria monocytogenes* leads to abnormal cell division and loss of actin-based motility. Infect Immun.

[20] Raffelsbauer D, Bubert A, Engelbrecht F, Scheinpflug J, Simm A, Hess J, Kaufmann SH, Goebel W (1998). The gene cluster inlC2DE of *Listeria monocytogenes* contains additional new internalin genes and is important for virulence in mice. Mol Gen Genet.

[21] Sabet C, Lecuit M, Cabanes D, Cossart P, Bierne H (2005). LPXTG protein InlJ, a newly identified internalin involved in *Listeria monocytogenes* virulence. Infect Immun.

[22] Smith K, Youngman P (1992). Use of a new integrational vector to investigate compartment-specific expression of the *Bacillus subtilis* spoIIM gene. Biochimie.

[23] Stachowiak R, Wisniewski J, Osinska O, Bielecki J (2009). Contribution of cysteine residue to the properties of *Listeria monocytogenes* listeriolysin O. Can J Microbiol.

[24] Varma A, Young KD (2004). FtsZ collaborates with penicillin binding proteins to generate bacterial cell shape in *Escherichia coli*. J Bacteriol.

